# Ammoniacal nitrogen recovery from swine slurry using a gas-permeable membrane: pH control strategies and feed-to-trapping volume ratio

**DOI:** 10.1007/s11356-024-32193-5

**Published:** 2024-02-20

**Authors:** Andreu Serra-Toro, Yasmina Ben Hammou Abboud, Maria Alicia Cardete-Garcia, Sergi Astals, Francesco Valentino, Francesc Mas, Joan Dosta

**Affiliations:** 1 Chemical Engineering and Analytical Chemistry Department, University of Barcelona, Barcelona, Catalonia Spain; 2 Materials Science and Physical Chemistry Department & Research Institute of Theoretical and Computational Chemistry (IQTCUB), University of Barcelona, Barcelona, Catalonia Spain; 3 Water Research Institute, University of Barcelona, Barcelona, Catalonia Spain; 4 Department of Environmental Sciences, Informatics and Statistics, Ca’ Foscari University of Venice, Mestre-Venice, Italy

**Keywords:** Ammonia recovery, Ammonium sulphate, Fertiliser, Membrane technology, Pig slurry

## Abstract

Gas-permeable membrane (GPM) technology is gaining interest to recover nitrogen from residual effluents due to its effectiveness, simple operation and capacity of producing a nutrient rich product with fertilising value. In this study, a GPM contactor was used at 25 °C to recover total ammoniacal nitrogen (TAN) from swine slurry as a concentrated (NH_4_)_2_SO_4_ solution. Firstly, a synthetic solution was tested on a wide pH range (6–12). Results showed that the ammonia mass transfer constants (*K*_*m*_) increased from 7.9·10^−9^ to 1.2·10^−6^ m/s as the pH increased. The reagent consumption to control the pH per mole nitrogen recovered had a minimum at pH 9, which showed a *K*_*m*_ value of 3.0·10^−7^ m/s. Secondly, various pH control strategies were tested using swine slurry, including (i) no pH control, (ii) pH control at 8.5, 9.0 and 10.0, and (iii) an initial spike of the NaOH equivalent to the required to control the pH at 9. The test without pH control reached a TAN recovery of around 60%, which could be an interesting strategy when high nitrogen recoveries or short operating times are not required. The pH control at 9 stood out as the most favourable operating condition due to its high *K*_*m*_ and lower reagent consumption. Thirdly, several feed-to-trapping volume ratios ranging from 1:1 to 15:1 were tested using swine slurry at pH 9. These assays revealed that a GPM process with a high feed-to-trapping volume ratio fastens the recovery of 99% of TAN as a high purity (NH_4_)_2_SO_4_ solution containing 40 g N/L.

**Supplementary Information:**

The online version contains supplementary material available at 10.1007/s11356-024-32193-5.

## Introduction

Animal manure from intensive livestock farming must be properly managed to mitigate its environmental impacts. Excessive application of manure to the soil results in several environmental problems, such as the accumulation of contaminants in the soil, surface and groundwater contamination, and ammonia emissions (Morrissy et al. 2021). Catalonia is one of the European regions with the highest livestock density. 39.9% of Catalonia’s total surface has been declared vulnerable to contamination by nitrates from agricultural sources, affecting 49.2% of the Catalan municipalities (ACA 2023). Furthermore, the agriculture and livestock sectors contribute to 12% of the greenhouse gases emitted in Catalonia (Generalitat de Catalunya 2023). The agricultural sector is also responsible for most of the ammonia atmospheric emissions in the European Union (European Environment Agency, 2023), a source of environmental and health issues (Blaas and Kroeze 2016; Temkin et al. 2019). These emissions also represent a loss of ammonia, a valuable building block for the fertiliser and chemical industry. Ammonia fixation and fertiliser production are energy intensive processes; therefore, the world is experiencing a sharp price increase (Fertilizers Europe 2023). Accordingly, there is an urgent need to develop technologies that enable the recovery of nitrogen from manure whilst preventing the presence of other contaminants that may compromise their quality, such as heavy metals and pathogenic microorganisms. Such technologies would increase the availability of fertilisers, develop circular economy schemes and mitigate environmental issues related to the current manure management (Prenafeta-Boldú and Parera 2020).

Several technologies for total ammoniacal nitrogen (TAN) recovery from livestock manure have been studied, including ammonia capture via carbon-based and mineral adsorbents (Li et al. 2015), struvite precipitation (Zhang et al. 2020; Astals et al. 2021), ammonia stripping (Chen et al. 2023), electrodialysis (Ippersiel et al. 2012), microbial electrolysis cells (Zou et al. 2021), gas-permeable membrane (GPM) technology (Vanotti et al. 2017), membrane distillation (Zarebska et al. 2015) and membrane concentration and ion exchange (Lim et al. 2012). Munasinghe-Arachchige et al. (2021), who carried out a multicriteria-based analysis of some of the aforementioned technologies, concluded that GPM is the most suitable technology to recover TAN from sewage sludge anaerobic digestion reject water. The increasing interest on GPM contactors technology to recover TAN from residual effluents is related to its relatively simple operation, little energy consumption and the generation of a valuable fertiliser product (Darestani et al. 2017; Beckinghausen et al. 2020).

The GPM process for nitrogen recovery consists of circulating a nitrogen-rich effluent (feed solution) through one side of a selective hydrophobic membrane whilst circulating a trapping solution (e.g., diluted H_2_SO_4_) on the other side of the membrane. Hydrophobic membrane only allows the diffusion of neutral and non-hydrated molecules (e.g., free ammonia) through the membrane’s micropores. The driving force for ammonia mass transfer is the concentration gradient of unionised ammonia between the feed and the trapping solution (Lee et al. 2021). Therefore, those operating parameters that have a significant effect on NH_4_^+^/NH_3_ equilibrium have an impact on the efficiency of the process, such as pH and temperature (Rongwong and Goh 2020; Serra-Toro et al. 2022b). Several studies have shown that GPM contactor technology can reach TAN recovery efficiencies above 95% from a wide variety of residual effluents (Dube et al. 2016; Noriega-Hevia et al. 2020). Successful experiences have been reported when applying GPM contactors to recover TAN from swine slurry with a nitrogen content ranging from 1.0 to 9.9 g N/L (García-González et al. 2015; Garcia-González and Vanotti 2015; Daguerre-Martini et al. 2018; Riaño et al. 2019) and digested swine manure with 2.1 to 3.2 g N/L (Dube et al. 2016; Riaño et al. 2021). Some publications have successfully coupled GPM technology with other treatment units for slurry treatment, such as anaerobic bioreactors and electrodialysis (Molinuevo-Salces et al. 2018; Rivera et al. 2022; González-García et al. 2022).

Despite these successful results, further research is required to optimise GPM technology operating conditions and control strategies in order to find a compromise solution between transfer rates, reagent consumption, and membrane life-span. Without pH control, TAN recoveries between 57 and 66% have been reported for swine slurry (García-González et al. 2015; Garcia-González and Vanotti 2015). To obtain higher nitrogen recoveries and transfer rates, the pH of the feed solution is typically controlled at a certain set point. Most researchers have operated their GPM processes at pH values between 8 and 11, with higher transfer rates reported at pH above 10 (Garcia-González and Vanotti 2015; Munasinghe-Arachchige et al. 2020; Aguado et al. 2022). Nevertheless, Lee et al. (2021) suggested that moderate alkaline pH values (around 9) are preferable to prevent inorganic fouling by precipitates on the membrane. Additionally, Serra-Toro et al. (2022b) reported that pH 9 has a lower reagent consumption per mole of TAN recovered than pH 10 and 11.

Temperature and pH have been identified as the most important operating factors to be regulated for GPM technology due to their high influence on ammoniacal nitrogen speciation (Zhu et al. 2024). However, other operating parameters could also impact the efficiency of the process. One operating parameter that needs further research is the volume of trapping solution required to treat a certain volume of feed solution and its replacement frequency (Riaño et al. 2021; Sheikh et al. 2022). Due to higher marketability and market price, it is important to obtain a trapping solution with high concentration of nitrogen salt but without compromising the concentration gradient between both sides of the membrane. Molinuevo-Salces et al. (2020) reached a maximum TAN concentration of 32 g N/L in the trapping solution for swine slurry, whilst Riaño et al. (2021) reported a maximum concentration of 35 g N/L for digested swine manure. These nitrogen concentrations in the trapping solution are in line with the 28 and 37 g N/L reported by Oliveira Filho et al. (2018) and Daguerre-Martini et al. (2018), respectively.

The main objective of this study was to determine the suitability of different operation strategies to optimise the performance of GPM technology treating swine slurry at room temperature (25 °C) as well as to determine the quality of the concentrated (NH_4_)_2_SO_4_ trapping solution. Process efficiency was determined in terms of ammoniacal nitrogen recovery, nitrogen mass transfer constant and reagents consumption. Several feed solution pH values and pH control strategies were tested using a synthetic solution and pre-treated swine manure. The GPM contactor performance was also evaluated in single and/or multiple stage process using a feed-to-trapping volume ratio of 1:1, 10:1 and 15:1. The commercial value of the most concentrated (NH_4_)_2_SO_4_ trapping solution obtained was also evaluated by considering the presence of other species, such as metals, ions and organic matter.

## Materials and methods

### Synthetic feed solution and swine slurry composition and origin

Synthetic wastewater and the liquid fraction of swine manure (namely swine slurry) were used as feed solution in this study. The synthetic wastewater contained 1.8 g N/L (using NH_4_Cl from AppliChem) and 6.0 g/L of acetic acid (J.T. Baker). Three batches of swine manure were collected at a swine farm in Artesa (Catalonia, Spain). The swine slurry was obtained after centrifugation (16,000 × g, 8 min; Sigma 1–14 microcentrifuge) and filtration (1.2-μm cellulose filters). The liquid fraction was stored in a refrigerator at 4 °C until use. Table 1 summarises the main characteristics of the swine slurries used in this study.

**Table 1:** Characteristics of the swine slurry

Parameter	Units	Batch 1	Batch 2	Batch 3
pH	–	8.4 ± 0.1	8.1 ± 0.2	8.2 ± 0.2
Total solids	g TS/kg	13.2 ± 0.5	–	–
Volatile solids	g VS/kg	6.1 ± 0.4	–	–
Total COD	g COD/L	11.9 ± 0.1	18.7 ± 1.1	13.1 ± 0.2
Soluble COD	g COD/L	5.5 ± 0.2	14.2 ± 2.1	8.7 ± 0.3
Alkalinity	g CaCO_3_/L	10.0 ± 0.2	12.8 ± 0.3	11.1 ± 0.3
TAN	g N/L	2.3 ± 0.3	2.8 ± 0.1	2.7 ± 0.2

### Experimental set up

The experimental set up consisted of two sealed glass tanks and a microporous hollow-fibre polypropylene membrane contactor (3 M Company) with an active surface area of 0.50 m^2^. A 2-L tank was used for the trapping solution (diluted H_2_SO_4_), and a 5-L jacketed tank was used for the feed solution. Both the feed and the trapping solutions were pumped in closed loops through the membrane module using two peristaltic pumps (Masterflex L/S models 7518–10 and 7518–12, respectively). A flowrate of 15 and 5 L/h were used for the feed and the trapping solution, respectively. The feed solution velocity was higher to decrease the thickness of the liquid boundary layer (LBL) and increase the nitrogen flux (Sethunga et al. 2019). This effect is considered negligible for the trapping solution side LBL (Tan et al. 2006). The feed solution was recirculated through the hollow-fibre membrane shell side, and the trapping solution was recirculated internally (lumen side). Circulating the feed on the shell side has been reported to facilitate membrane cleaning and leads to higher TAN recovery efficiencies (Hasanoğlu et al. 2010). The hydrophobic nature of the membrane and its small pores prevented wetting due to the capillary effect (Boehler et al. 2015).

The tanks were equipped with magnetic stirrers (IKA C-MAG HS7) to keep the content well mixed. A water bath (Thermo Scientific HAAKE DC30) was used to maintain the feed solution at 25 °C. The pH set point of both solutions was controlled using pH-metres (Crison 53 35 electrodes connected to Crison pH 28 controllers) by the addition of H_2_SO_4_ 75% in the trapping solution and NaOH 10 M in the feed solution.

### Experiments methodology

Table 2 summarises the operating conditions of the three phases carried out in this study.

**Table 2:** Operating conditions of the experiments carried out in this study

Phase	1	2					3		
Wastewater	Synthetic wastewater	Swine slurry (Batch 1)					Swine slurry (Batch 2)		Swine slurry (Batch 3)
Test name	1A–1G	2A	2B	2C	2D	2E	3A	3B	3C
Feed pH value	6, 7, 8, 9, 10, 11, 12	8.5	9.0	10.0	No control	*	9.0	9.0	9.0
Number of stages	1	1	1	1	1	1	10	1	1
*V*_feed_/*V*_trapping_ (L/L)	2.0/2.0	2.5/0.5					0.5/0.5	5.0/0.5	6.0/0.4
Volume ratio	1:1	5:1					1:1	10:1	15:1
Duration (h)	8	15	15	15	35	15	40	25	16

*The total NaOH consumption of Test 2B was added at the beginning of the experiment

#### Phase 1: effect of pH on synthetic feed solution nitrogen recovery

Phase 1 experiments were carried out to determine the effect of the feed solution pH on membrane performance. Seven different pH values (6, 7, 8, 9, 10, 11 and 12) were tested whilst keeping the trapping solution pH below 2. The volumes of the synthetic feed and trapping solutions were 2 L each. The trapping solution had an initial H_2_SO_4_ concentration of 17.5 mM. These experiments were conducted in duplicate and run for 8 h. To monitor these experiments, a sample from the feed and the trapping solutions were withdrawn by duplicate from the tanks every 30 min during the first hour and every 60 min afterwards.

#### Phase 2: effect of pH control strategies on swine slurry nitrogen recovery

Tests 2A, 2B and 2C aimed to determine the effect of adjusting the pH of the swine slurry at pH 8.5, 9.0 and 10.0 on the membrane performance, respectively. These pH values were selected based on Phase 1 results. Test 2D was performed without pH control in the feed tank to determine the feasibility of a strategy that did not consume any NaOH. Test 2E was designed to determine the effect of adding at the beginning of the experiment all the alkali required to maintain the feed pH at 9.0 and completely recover TAN (based on Test 2B results). In this Phase, the feed (swine slurry from Batch 1) and trapping solution volumes were 0.5 and 2.5 L, respectively. To monitor these experiments, samples of both feed and trapping solutions were withdrawn by duplicate from both tanks every 30 min during the first 1.5 h and afterwards every 60 min.

#### Phase 3: effect of feed-to-trapping volume ratio on swine slurry nitrogen recovery

These experiments aimed to determine the effect of a single or multiple-stage configuration on process performance for a 10:1 volume ratio between the feed and the trapping solution. These experiments were conducted using the swine slurry from Batch 2 at pH 9.0. Test 3A consisted of 10 stages where the feed-to-trapping volume ratio of each stage was 1:1. The volume of both solutions in each stage was 0.5 L. The trapping solution was reused for the subsequent stages whilst 0.5 L of fresh swine slurry replaced the nitrogen-spent swine slurry of the previous stage. In Test 3A, the trapping solution was highly concentrated (H_2_SO_4_ 1.5 M) since it was not replaced during the 10 stages. Test 3B consisted of a single-stage process with a feed-to-trapping volume ratio of 10:1, using 5 L of swine slurry and 0.5 L of trapping solution (H_2_SO_4_ 70 mM).

Test 3C aimed to obtain a highly concentrated (NH_4_)_2_SO_4_ trapping solution as well as to quantify the presence of contaminants that could compromise its commercial value. To reach a 20 wt.% (NH_4_)_2_SO_4_ concentrated solution the feed-to-trapping volume ratio was set at 15:1. More concisely, the swine slurry (Batch 3) volume and the trapping solution volume were 6.0 L and 0.4 L, respectively. The experiment lasted 16 h and was carried out at pH 9.0. The resulting trapping solution was exhaustively analysed to determine the presence of contaminants such as metals, ions and total organic carbon (TOC).

To monitor the experiments of Phase 3, duplicate samples from the feed tank were withdrawn every 30 min during the first 4 h and afterwards every 60 min. However, the frequency of sampling in the trapping solution was reduced (from 30 to 60 min during the first 4 h and from 30 to 120 min afterwards) when higher feed-to-trapping volume ratios were tested to minimise its impact in the results of the trapping solution characterisation.

### Analytical methods

An ammonium electrode (Thermo Scientific, 9512HPBNWP) was used to quantify the TAN concentration present in the feed and trapping solution samples following the procedure 4500-NH3D (APHA 2017). Total and soluble COD were determined following the Standard Method 5220C (APHA 2017). Total solids (TS) and volatile solids (VS) were determined according to the Standard Method 2540G (APHA 2017). Alkalinity was determined according to the Standard Method 2320B, using an automated titrator (Crison pH Burette 24) with HCl 0.1 M and a pH endpoint of 4.30. Acetic acid was analysed using a Shimadzu GC-2010 plus gas chromatograph equipped with an Agilent DB-FFAP capillary column and flame ionisation detector. Heavy metals (As, Zn, Pb, Cd, Hg, Cu, Mn, and Li) and other elements (S, K, Mg, Ca, Na, Fe, and P) concentration were determined using an ICP-MS spectrophotometer (Perkin Elmer Nexion 350D). Before ICP-MS analysis, the pig slurry was digested (1 mL sample + 3 mL HNO_3_ + 1 mL H_2_O_2_) in a closed Teflon reactor at 90 °C for 24 h. Total Carbon (TC) was determined using a Multi N/C 3100 Analytik Jena by a catalytic combustion process where the resulting CO_2_ is quantified by an infrared detector. Inorganic carbon (IC) analysis is carried out by the injection of the sample to a phosphoric acid and quantifying the CO_2_. TOC was the difference between TC and IC.

### Calculations and statistical analysis

The TAN removal and recovery efficiencies were determined using Eqs. 1 and 2, respectively. The TAN mass (g) is the following: TAN_*f*_(0) for the feed solution at the beginning, TAN_*f*_(*t*) for the feed solution at a specific time, TAN_*t*_(0) for the trapping solution at the beginning and TAN_*t*_(*t*) for trapping solution at a specific time. The difference between TAN removal and recovery is associated with TAN losses.

1 $$\%\;\mathrm{TAN}\;\mathrm{removal}\;\left(t\right)=\frac{{\text{TAN}}_f\left(0\right)-{\text{TAN}}_f(t)}{{\text{TAN}}_f(0)}\cdot100$$

2 $$\%\;\mathrm{TAN}\;\mathrm{recovery}\;\left(t\right)=\frac{{\text{TAN}}_t\left(t\right)-{\text{TAN}}_t(0)}{{\text{TAN}}_f(0)}\cdot100$$

The ammonia mass transfer constant (*K*_*m*_) was used to evaluate the ammonia flux through the membrane (Reig et al. 2021; Serra-Toro et al. 2022a, 2022b). This parameter quantifies the ammonia transfer under specific conditions. Equation 3 determines the *K*_*m*_ value [m/s] from experimental concentration data if the NH_3_/NH_4_^+^ equilibrium is fulfilled during the operation. In Eq. 3, *c*_TAN,*f*_(*t*) is the concentration of TAN in the feed solution at any time [g N/L], *c*_TAN,*f*_(0) is the initial concentration of TAN in the feed solution [g N/L], *A* is the area of the membrane [m^2^], *V*_*f*_ is the volume of the feed solution [m^3^] and t is the time [s]. The *K*_*m*_ depends on the pH and temperature of the feed solution, amongst other operating factors.

3 $$\frac{{C}_{{\text{TAN}},f}(t)}{{C}_{{\text{TAN}},f}(0)}={\text{exp}} \left(\frac{-{K}_{m} A}{{V}_{f}} t\right)$$

This model was coded in Python using the curve fit function of the SciPy and the Levenberg–Marquardt algorithm to perform non-linear least squares estimates. The algorithm estimates *K*_*m*_ and its standard deviation by fitting the TAN concentration of the feed and trapping solution in the tanks.

The reagent consumption was calculated in moles of reagent (NaOH and H_2_SO_4_) per mole of TAN recovered to facilitate tests’ comparison. The total consumption accounted for the total amount of reagent added throughout the test, whereas the consumption to control the pH did not consider the initial addition to reach the pH set point.

The Fisher test (*F*-test) was used to compare the variances of the *K*_*m*_ values. The variances were obtained as the square of the standard deviation. In the *F-*test, an *F*-calculated ratio between the two variances is compared against an *F*-table value, dependent on both experiments’ degrees of freedom and assuming a confidence interval of 99.5%. Whenever these variances presented homogeneity, the Student *t*-test was applied to compare the *K*_*m*_ values. For this purpose, a *t*-calculated ratio based on the *K*_*m*_ values, variances and number of experimental observations was compared to a *t*-table value, dependant on the degrees of freedom and assuming a 99.5% confidence interval. If the variances were non-homogeneous, the Welch correction was introduced in the Student *t*-test.

## Results and discussion

### Effect of pH on the membrane performance for synthetic feed solution (Phase 1)

**Fig. 1:**
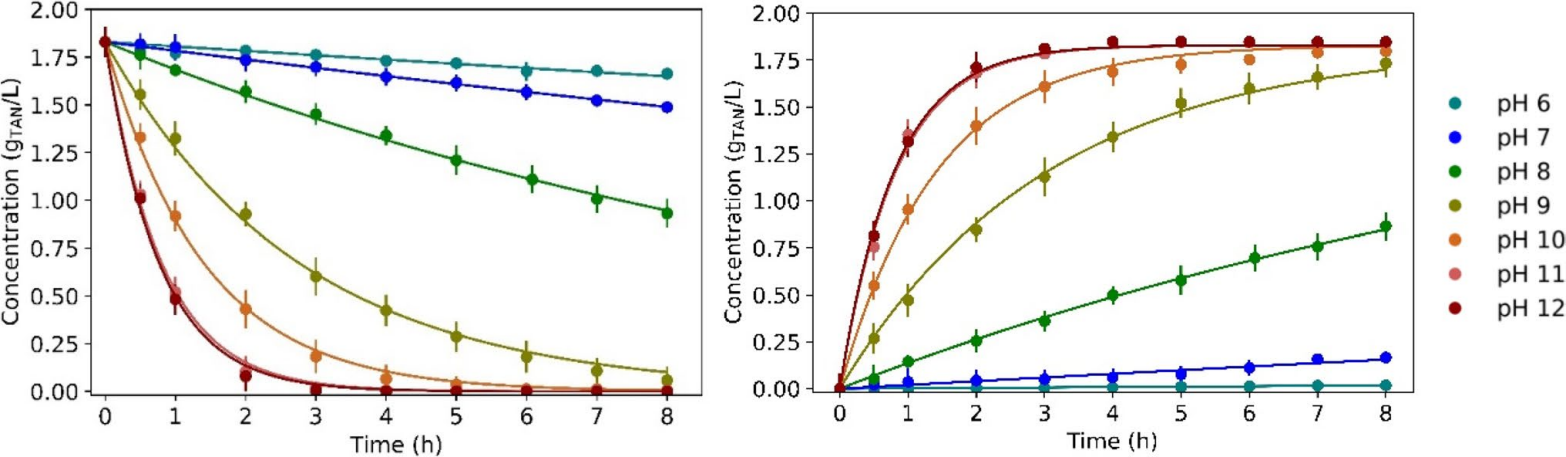
TAN concentration of the (left) feed solution and (right) trapping solution of the experiments carried out at different synthetic feed solution pH (Phase 1). Dots represent the experimental data and solid lines represent their modelled TAN concentration profile

The variation of TAN concentration in the feed and trapping solutions at different pH values is illustrated in Fig. 1. The nitrogen transfer was highly dependent on the feed solution pH since pH determines the fraction of ammoniacal nitrogen present as free ammonia at a certain temperature and feed solution composition (ionic strength). For the experiments working at pH 11 and 12, the TAN concentration in the feed solution decreased considerably during the first 2 h (< 0.1 g N/L). On the other hand, the calculated ammonia mass transfer constant (*K*_*m*_) sharply decreased for experiments carried out at pH values below 9. An almost complete TAN recovery (> 95%) was achieved for tests at pH values from 9 to 12 after 8 h. In all the tests, acetic acid was never detected in the trapping solution since only unionised acetic acid could pass through microporous hydrophobic membranes (Aydin et al. 2018).

The calculated K_m_ values from the synthetic feed solution experiments at 25 °C are shown in Table 3. The pH had a statistically significant effect on the *K*_*m*_ values from pH 6 to 11. However, no statistically significant difference was observed between the *K*_*m*_ at pH 11 and 12, since the NH_3_ percentage of TAN is almost 100% at these pH values. The calculated K_m_ values increased with pH, reaching a high *K*_*m*_ of (3.0 ± 0.1)·10^−7^ m/s at pH 9, and being the maximum *K*_*m*_ (1.15 ± 0.05)·10^−6^ m/s at pH 11 and 12, which are in agreement with the results reported by Vecino et al. (2019) and Reig et al. (2021) when working with a hollow-fibre polypropylene membrane contactor.

**Table 3:** TAN recovery and removal efficiencies, *K*_*m*_ values and reagents consumption in Phase 1 experiments (average values ± standard deviation)

Test	pH of feed solution	TAN removal/recovery at 8 h	*K*_*m*_	Total alkali consumption	Alkali consumption (pH control)	Total acid consumption	Acid consumption (pH control)
	Units	%	m/s	mol NaOH/mol TAN recovered		mol H_2_SO_4_/mol TAN recovered	
1A	6	12.0/1.1	(7.9 ± 6.7)·10^−9^	41 ± 13	2.3 ± 1.2	12.0 ± 5.2	-
1B	7	16.3/10.1	(1.6 ± 0.9)·10^−8^	4.29 ± 0.54	0.86 ± 0.14	1.13 ± 0.05	-
1C	8	49.9/49.2	(7.1 ± 0.3)·10^−8^	1.62 ± 0.02	0.83 ± 0.08	0.65 ± 0.03	0.39 ± 0.13
1D	9	97.8/97.8	(3.0 ± 0.1)·10^−7^	1.28 ± 0.17	0.75 ± 0.24	0.54 ± 0.08	0.67 ± 0.07
1E	10	99.7/99.6	(6.7 ± 0.1)·10^−7^	1.39 ± 0.30	0.37 ± 0.03	0.78 ± 0.07	0.62 ± 0.06
1F	11	99.8/99.8	(1.2 ± 0.1)·10^−6^	1.51 ± 0.19	0.03 ± 0.02	0.71 ± 0.04	0.59 ± 0.04
1G	12	99.9/99.9	(1.2 ± 0.1)·10^−6^	1.30 ± 0.08	-	0.69 ± 0.03	0.57 ± 0.03

The total reagent consumption per mole of TAN recovered for both NaOH and H_2_SO_4_ was statistically similar for those tests at or above pH 8 and noticeably higher for tests at pH 6 and 7 (due to the low nitrogen recovery under these pH values). The lowest specific consumptions were obtained at pH 9 because, at higher pH values, the higher reagent addition did not compensate the increase in nitrogen flux through the membrane. The NaOH consumption per mole of TAN recovered to control the pH decreased as the pH increased. The H_2_SO_4_ consumption to control the pH at 6 and 7 was null since no acid addition was necessary due to the low TAN recovery. It is worth highlighting that both reagent consumptions were close to the stoichiometric values, i.e., 1 mol of NaOH and 0.5 mol of H_2_SO_4_ per each mole of nitrogen that crossed the membrane.

From these experiments results, the optimum feed solution pH was considered 9 because (i) it provided a high *K*_*m*_ and (ii) a reagent consumption was close to stoichiometry. Furthermore, according to Lee et al. (2021), operating at moderately alkaline pH values is favourable for preventing fouling on the membrane caused by salts precipitation.

**Fig. 2:**
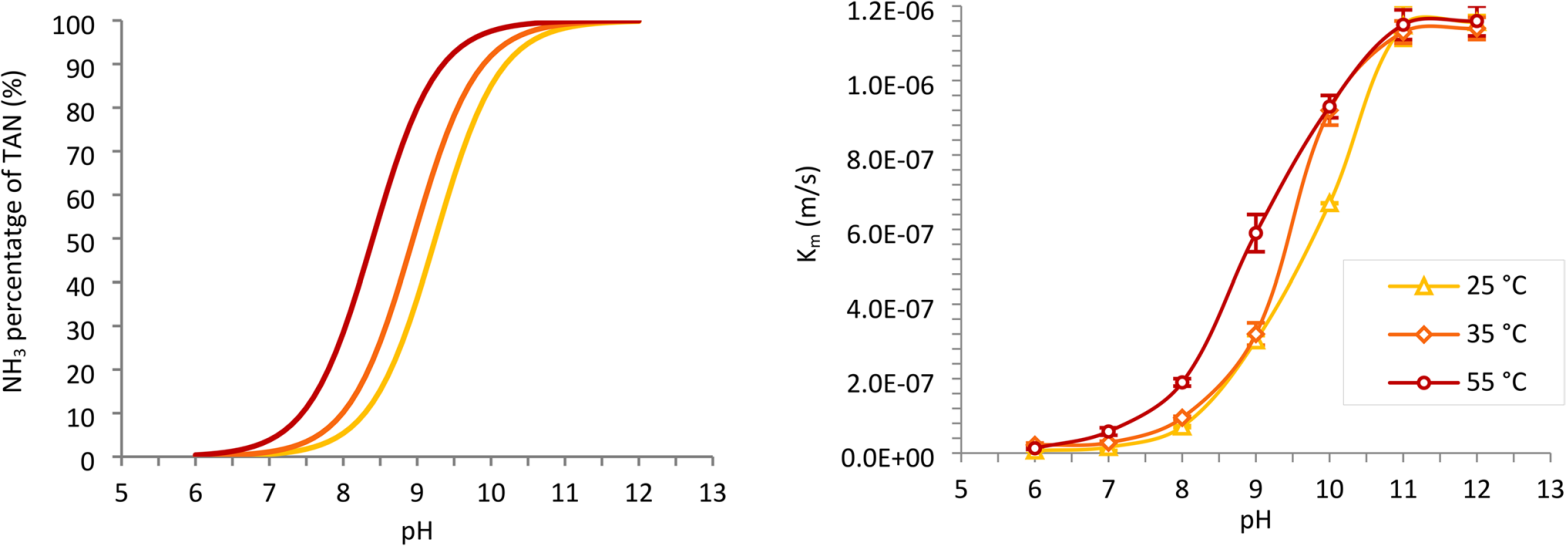
NH_3_ percentage of TAN (left) and average ammonia mass transfer constant (K_m_) at different temperatures (25, 35 and 55 °C) and pH values (right)

Figure 2 (right) combines the *K*_*m*_ values obtained in this publication at 25 °C with those obtained in our previous publication at 35 and 55 °C using the same membrane module and similar operating conditions (Serra-Toro et al. 2022b). The *K*_*m*_ values at 25 °C had a similar order of magnitude compared those at 35 and 55 °C. Regardless of the temperature, for pH values above 11 more than 98% of the TAN is in the form of NH_3_ and *K*_*m*_ reached a maximum value. Thus, further increasing the pH or temperature would not lead to any significant improvement on *K*_*m*_. At pH 6, less than 1% of TAN is present in the form of NH_3_ which explains the lower *K*_*m*_ values. For those intermediate pH values, the effect of temperature represents an increase in the *K*_*m*_ (see values in Online Resource 1). This is caused by a decrease in the NH_4_^+^/NH_3_ pKa when temperature increases, which results in a higher NH_3_ fraction of the TAN in the feed solution. Figure 2 (left) shows the NH_3_ percentage of TAN for the studied pH and temperature conditions, considering their effect on the NH_4_^+^/NH_3_ acid–base constant and an ideal solution (Anthonisen et al. 1976).

### Effect of pH control strategies on swine slurry nitrogen recovery (Phase 2)

**Fig. 3:**
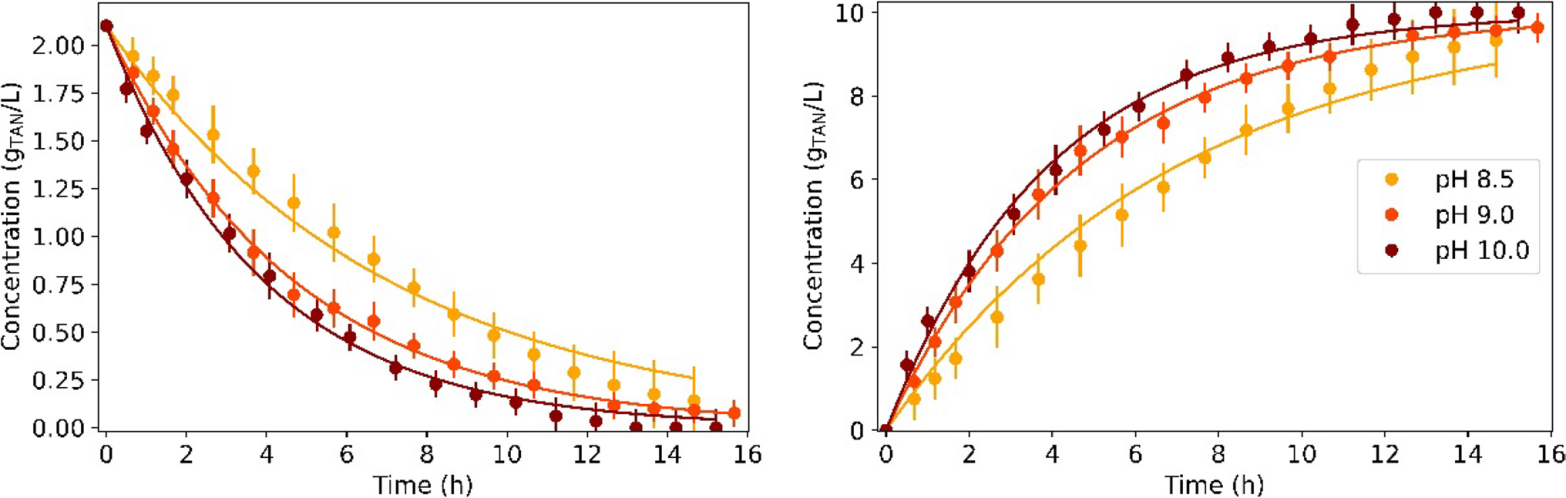
TAN concentration of the (left) feed and (right) trapping solution of the experiments carried out using swine slurry at pH 8.5, 9.0 and 10.0. Dots represent the experimental data and solid lines represent their modelled TAN concentration profile

Figure 3 shows the TAN concentration evolution in the feed and trapping solutions of the tests carried out at pH of 8.5, 9.0 and 10.0 using swine slurry (Batch 1). Similar to the results obtained with the synthetic solution (Sect. 3.1), the nitrogen transfer rate enhanced with increasing pH values. The calculated *K*_*m*_ was (1.98 ± 0.07) ·10^−7^, (2.99 ± 0.04) ·10^−7^ and (3.56 ± 0.09)·10^−7^ m/s for pH 8.5, 9.0 and 10.0, respectively (Table 4). These results showed a higher improvement in membrane performance when raising the pH from 9.0 to 10.0 than from 8.5 to 9.0. The TAN removal increased from 84.0 to 96.3% and to 98.1% as the feed solution pH increased from 8.5 to 9.0 and to 10.0, respectively. These results showed a noticeably lower performance of the membrane at pH 8.5. The statistical analysis showed that the *K*_*m*_ values obtained were statistically different for each pH condition.

**Table 4:** TAN recovery and removal, K_m_ value and reagent consumption of the experiments carried out with swine slurry, including Phases 2 and 3 (average values ± standard deviation)

Test	Test conditions	TAN removal /recovery at final time	*K*_*m*_	Total alkali consumption	Alkali consumption (pH control)	Total acid consumption	Acid consumption (pH control)
		%	m/s	mol NaOH/mol TAN recovered		mol H_2_SO_4_/mol TAN recovered	
2A	pH 8.5	93.3/84.0	(1.98 ± 0.07) ·10^−7^	2.03	1.54	1.03	0.76
2B	pH 9.0	96.4/96.3	(2.99 ± 0.04) ·10^−7^	0.55	0.32	0.62	0.55
2C	pH 10.0	98.1/98.1	(3.56 ± 0.09) ·10^−7^	0.87	0.44	0.69	0.59
2D	No pH control	65.6/58.4	(4.34 ± 0.05) ·10^−8^	0.00	0.00	1.23	1.08
2E	Alkali spike	98.0/98.0	(4.46 ± 0.09) ·10^−7^	0.58	0.00	0.70	0.44
3A	10 stages	99.8/99.6	(4.05 ± 0.15)·10^−7^	0.68	0.54	0.73	0.00
3B	1 stage	99.2/99.2	(3.74 ± 0.19)·10^−7^	0.88	0.86	0.63	0.60
3C	1 stage	99.2/99.1	(1.30 ± 0.18)·10^−7^	1.04	0.65	0.65	0.40

The statistical analysis revealed that the *K*_*m*_ values obtained were statistically different for each pH condition. The *K*_*m*_ was also statistically different when comparing the synthetic solution tests with the swine slurry tests at the same pH. At pH 10, the swine slurry test had a *K*_*m*_ of (3.6 ± 0.09) ·10^−7^ m/s, lower than the synthetic wastewater *K*_*m*_ of (6.7 ± 0.1) ·10^−7^ m/s. However, this value did not noticeably vary when operating at pH 9. The lower *K*_*m*_ obtained with swine slurry could be caused by a more complex matrix of this high-strength feed solution, since ammonia speciation and solubilization are affected by the presence of organic matter and other ionic species, amongst other factors (Gonzalez-Salgado et al. 2023).

The NaOH consumption varied more than in synthetic feed solution experiments (Test 1A–1G) due to the higher alkalinity of the swine slurry. The amount of NaOH required to increase the swine slurry pH from 8.4 to 9.0 and 10.0 was 0.55 and 0.87 g NaOH/L, respectively. At pH 8.5, the lowest nitrogen transfer led to the highest reagent consumption per mole of TAN recovered (Table 4). Therefore, a controlled feed pH at 9 stands out as favourable operating condition when nearly complete TAN recovery is needed.

**Fig. 4:**
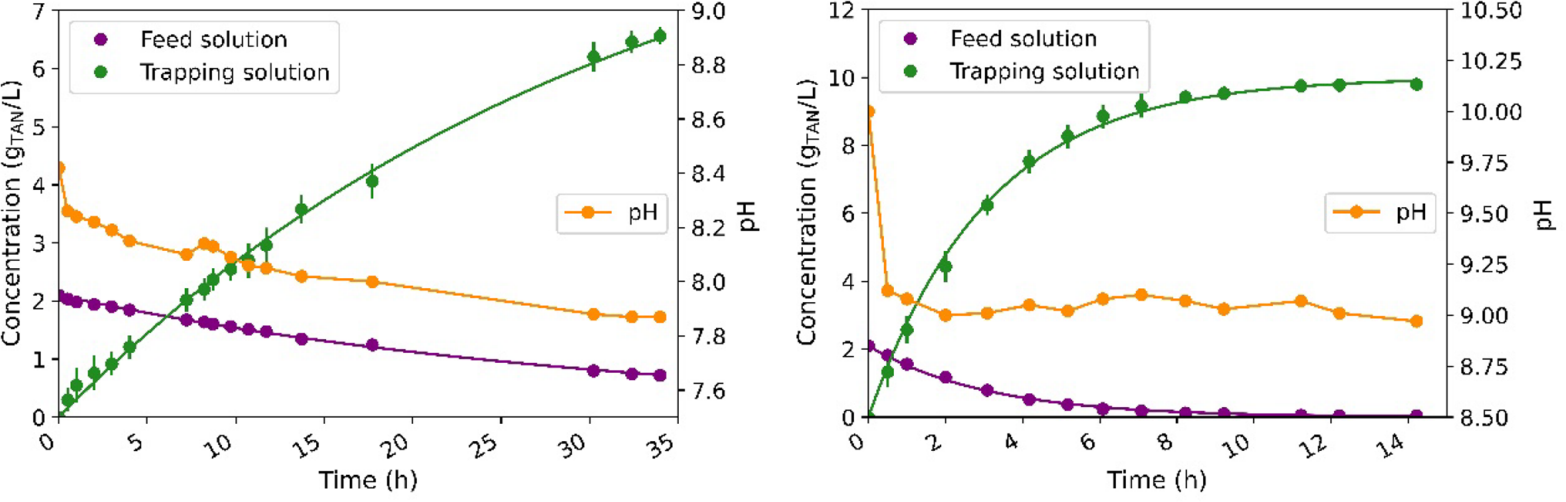
TAN concentration and pH evolution for (left) Test 2D and (right) Test 2E. Dots represent the experimental data and solid lines represent their modelled TAN concentration profile

The experiment carried out without pH control (Test 2D) is shown in Fig. 4 (left). The initial pH of the feed solution was 8.4 and it dropped to 7.8 after 35 h of operation. During the experiment, the TAN flux decreased progressively with pH because K_m_ is affected by the pH of the feed solution. In Test 2D, the average *K*_*m*_ was 4.34·10^−8^ m/s. The TAN recovery efficiency was 58.4%, a result consistent with similar tests carried out by Garcia-González and Vanotti (2015) and García-González et al. (2015). This experiment proved that TAN recovery can be achieved without consuming any alkali reagent, which could be useful when short operating times are not required and TAN recoveries about 60% are sufficient.

In test 2E, all the alkali reagent used in the pH 9 test (Test 2B) was added at the beginning of the experiment (i.e., 14.7 g of NaOH). As shown in Fig. 4 (right), the initial pH rose to near 10 after NaOH addition and decreased to 9 when swine slurry TAN concentration was almost 0. The TAN recovery at 15 h was higher than the observed in test at pH 9 (from 94.2 to 98.0%) since the pH until this moment was higher. Indeed, the average *K*_*m*_ increased from (2.99 ± 0.04)·10^−7^ to (4.46 ± 0.01)·10^−7^ m/s. The final H_2_SO_4_ consumption was also approximately the same as in Test 2B because both experiments spent the same amount of NaOH and obtained similar TAN recoveries.

Operating at high pH values results in highly competitive TAN recoveries, however, one critical factor to consider is the formation of precipitates at highly alkaline pH, particularly at pH values above 9 (Lee et al. 2021). In our case, 0.54 and 0.19 g SS/L were formed in tests performed at pH 10 in Test 2C and 2E, respectively. That entails the threat of severe membrane fouling issues under long term operation (Zarebska et al. 2015; Chen et al. 2023) and, consequently, the pH regulation at 9 was selected as the most favourable condition (amongst the pH control strategies tested) for the GPM treatment of swine slurry at 25 °C for nearly complete TAN recovery.

### Effect of feed-to-trapping volume ratio on swine slurry nitrogen recovery (Phase 3)

In Test 3A, the trapping solution was reused in each stage, allowing TAN accumulation through the 10 stages performed. In each stage, TAN was completely recovered in less than 4 h, much faster that in Test 2B where about 15 h were needed to completely recover the TAN. The faster TAN recovery is mainly due to the lower feed and trapping volumes (0.5 L) in this experiment compared to Test 2B volumes (2.0 L). The TAN recovery efficiency was 99.6% at the end of the experiment and the trapping solution had a concentration of 27 g N/L. This test resulted in a slight increase in TAN recovery compared to Test 2B (same pH value). The calculated *K*_*m*_ was (4.05 ± 0.15)·10^−7^ m/s, slightly higher than that obtained in Test 2B (2.99 ± 0.04)·10^−7^, which could be attributed to the different swine slurry collection batch. The total reagent consumption was 0.68 and 0.73 mol reagent/mol TAN recovered for the NaOH and H_2_SO_4_, respectively; slightly higher than those obtained in Test 2B.

Test 3B needed less time to completely recover TAN than Test 3A (15 vs. 35 h). However, no significant difference was encountered when comparing the experiments K_m_ values, i.e., (4.05 ± 0.15)·10^−7^ and (3.74 ± 0.19)·10^−7^ m/s for Test 3A and 3B, respectively, since both tests were carried out under the same pH and temperature. Therefore, these values show that the number of stages and the feed-to-trapping volume ratio have no significant effect on *K*_*m*_, unlike pH or temperature. It is worth considering that the calculated *K*_*m*_ values are related to shape of TAN concentration curve, which is similar in each stage for both tests. However, more stages led to larger operation times to obtain the same TAN recovery because the flat part of the curve (when the concentration gradient is lower) is repeated several times. Therefore, a high feed-to-trapping volume ratio is recommended to save time of operation, since the operation of high feed volumes takes advantage of the higher driving force (the difference of NH_3_ concentration in both sides of the hydrophobic membrane) throughout the entire operation period.

**Fig. 5:**
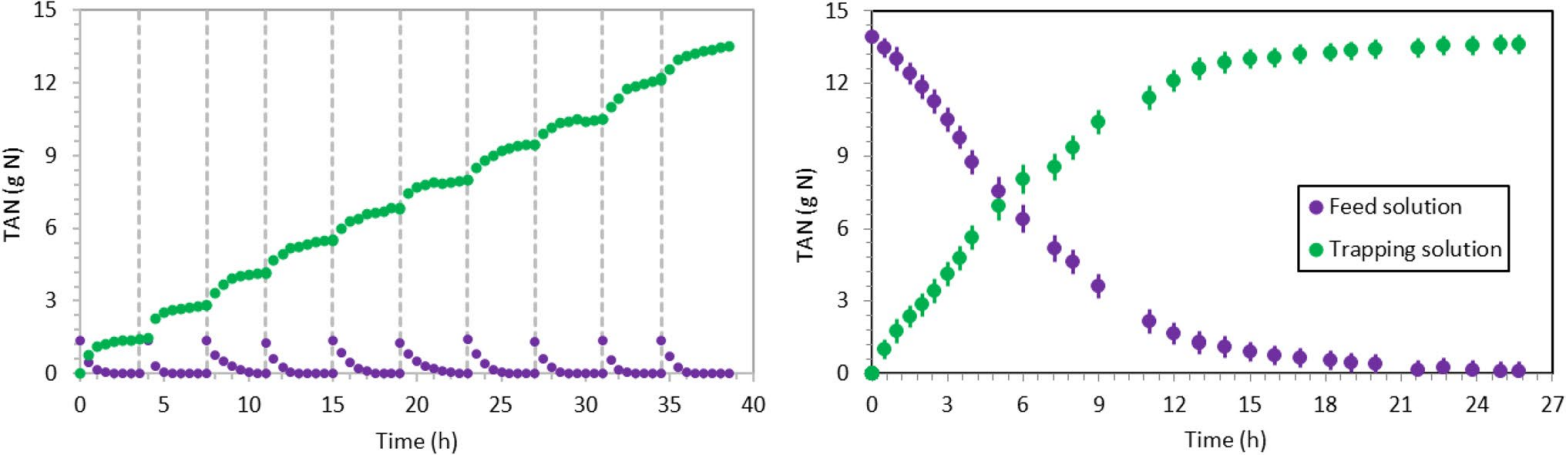
TAN mass in swine slurry and in the trapping solution in Test 3A (left), where 10 stages at 1:1 feed-to-trapping volume ratio were performed, and in Test 3B (right), where 1 stage at 10:1 feed-to-trapping volume ratio was assayed. Discontinuous lines represent the end and start of a new stage

In Test 3B, the final TAN concentration in the trapping solution was 27 g N/L as in Test 3A. The reagent consumption was also very similar to Test 3A (Table 4). However, for applications targeting high TAN recoveries, these results suggest that using a higher feed-to-trapping volume ratio in 1 stage require less operation time periods than when operating with multiple stages at lower feed-to-trapping volume ratios. These results could be explained by the deceleration of nitrogen transfer when lower TAN concentrations in the feed solution were reached (Fig. 5). Consequently, using a high feed volume takes advantage of the resulting higher average driving force and ammonia transfer rates.

When another swine slurry (Batch 3) was treated with a feed-to-trapping volume ratio of 15:1 in Test 3C, a TAN recovery of 99% was reached in 14 h and resulted in trapping solution with a concentration of 40.1 ± 0.1 g N/L. To the best of the authors’ knowledge, this TAN concentration in the trapping solution is within the upper range of TAN concentration values reported for GPM contactors (Vanotti and Szogi 2011; Oliveira Filho et al. 2018; Daguerre-Martini et al. 2018; Riaño et al. 2021). The calculated *K*_*m*_ value was (1.30 ± 0.18)·10^−7^ m/s, lower than that obtained in Test 2B ((2.99 ± 0.04) ·10^−7^ m/s), 3A ((4.05 ± 0.15)·10^−7^ m/s) and 3B (and (3.74 ± 0.19)· 10^−7^ m/s) carried out at the same pH and temperature conditions. The lower *K*_*m*_ could be caused by the different swine slurry batch. The H_2_SO_4_ consumption was close to that obtained in Test 3B whilst the specific alkali consumption was higher in Test 3C, probably due to the higher alkalinity of the swine manure (Batch 3 vs. Batch 2).

**Fig. 6:**
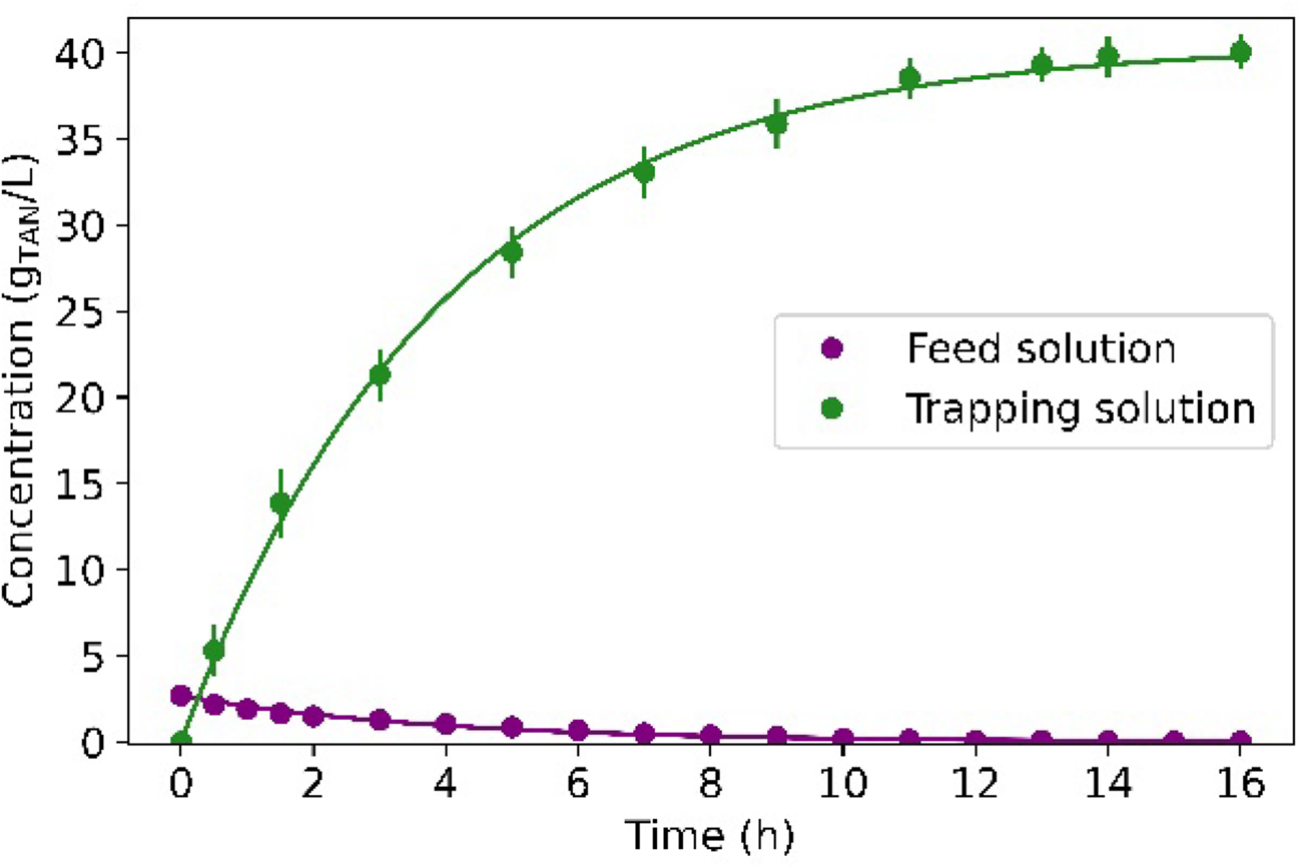
TAN concentration in swine slurry and in the trapping solution in Test 3C. Dots represent the experimental data and solid lines represent their modelled TAN concentration profile

Ammonium sulphate could be commercialised in the chemical industry at concentrations around 20 ± 0.5 wt.% in weight. The obtained concentration of 40.1 g N/L represents a concentration of (NH_4_)_2_SO_4_ of 18.9 wt.%. These results imply that GPM contactors could produce a trapping solution useful at commercial scale without needing further concentration processes. Moreover, the ammonium sulphate solution obtained from the GPM process had a very low concentration of contaminants, including metals and organic compounds. All the analysed metals were not detected in the trapping solution (detection limit of 100 ppb). The concentration of K, Ca, Mg, Fe, and P ions in the trapping solution was also below the detection limit (3 mg/L). These results are in agreement with those recorded by Riaño et al. (2019) who only observed 28 mg/L of K and did not detect Mg, Ca, Zn, Cu and Fe in the trapping solution (19 g N/L) of a GPM process for the treatment of swine manure. TOC analysis showed that some organic molecules diffused across the membrane since 3.2 ± 0.3 ppm were detected in the trapping solution after 16 h of operation. It is hypothesised that TOC diffusion provided a pale-yellow tone to the trapping solution, which occurred at the very end of the experiment based on visual observation. IC analyses showed that inorganic carbon did not diffuse across the membrane. To sum up, for almost complete TAN recovery of swine slurry at 25 °C, the results of this study suggest as preferred working conditions a pH control around 9 in the feed tank and the selection of a feed-to-trapping volume ratio that promotes 20 wt.% of (NH_4_)_2_SO_4_ in the trapping solution in 1 stage. These operating conditions would not only lead to high TAN recovery efficiencies, but also to the production of a concentrated (NH_4_)_2_SO_4_ trapping solution with high purity Fig. 6.

## Conclusions

The recovery of TAN from swine slurry was successfully performed using a lab-scale gas-permeable membrane (GPM) contactor. Tests carried out at different temperature and pH conditions revealed a correlation between the *K*_*m*_ values with the percentage of NH_3_ in TAN, which is related to the acid–base equilibrium. An increase of the *K*_*m*_ with pH was observed, reaching a maximum value of (1.2 ± 0.1)·10^−6^ m/s at pH 11 and 12 for temperatures between 25 and 55 °C. For the treatment of swine slurry using a GPM contactor at 25 °C, a TAN recovery of 58% was reached without controlling the pH in the feed tank. However, higher TAN recovery efficiencies would only be feasible by controlling the pH at values above 8.5. A pH control at 9 in the feed tank (*K*_*m*_ of (3.0 ± 0.1)·10^−7^ m/s) was considered suitable to diminish the reagent consumption per mass of TAN recovered and to minimise the membrane fouling threat, although higher *K*_*m*_ values could be achieved under more alkaline conditions. To reach TAN recovery efficiencies above 99% for the treatment of swine slurry at 25 °C, this study suggest the use of a 1 stage GPM process with a high feed-to-trapping volume ratio (in this study 15:1) that would also lead to the production of a highly (NH_4_)_2_SO_4_ concentrated trapping solution (18.9 wt.%) with high purity.

## Supplementary Information

Below is the link to the electronic supplementary material.

Supplementary file1 (DOCX 16 KB)
