# Supplementary material for: Ammoniacal nitrogen recovery from swine slurry using a gas-permeable membrane: pH control strategies and feed-to-trapping volume ratio
Source: Environ Sci Pollut Res Int. 2024 Feb 20;32(48):27625–36. doi: 10.1007/s11356-024-32193-5 (PMC12696102; doi:10.1007/s11356-024-32193-5)
Supplement: Supplementary file 1 — Supplementary file1 (DOCX 16 KB) [file 11356_2024_32193_MOESM1_ESM.docx]

**Ammoniacal nitrogen recovery from swine slurry using a gas-permeable membrane: pH control strategies and feed-to-trapping volume ratio**

Andreu Serra-Toro^1,2^, Yasmina Ben Hammou Abboud^1^, Maria Alicia Cardete-Garcia^1^, Sergi Astals^1^, Francesco Valentino^4^, Francesc Mas^2^, Joan Dosta^1,3,*^

^1^ Chemical Engineering and Analytical Chemistry Department. University of Barcelona, Barcelona, Catalonia, Spain.

^2^ Materials Science and Physical Chemistry Department & Research Institute of Theoretical and Computational Chemistry (IQTCUB), University of Barcelona, Barcelona, Catalonia, Spain.

^3^ Water Research Institute, University of Barcelona, Barcelona, Catalonia, Spain.

^4^ Department of Environmental Sciences, Informatics and Statistics, Ca’ Foscari University of Venice, Mestre-Venice, Italy

* Corresponding author e-mail: [jdosta@ub.edu](mailto:jdosta@ub.edu)

**Environmental Science and Pollution Research**

**Supplementary material**

**Online Resource 1.** K_m_ values for experiments carried out with synthetic solution as Feed (1A – 1G) at different pH and temperature values and their associated errors.

| K_m_ (m/s) | | | | | | |
| --- | --- | --- | --- | --- | --- | --- |
| pH | 25 ^o^C | | 35 ^o^C | | 55 ^o^C | |
|  | value | error | value | error | value | error |
| 6 | 6.67·10^-9^ | 7.88·10^-9^ | 2.20·10^-8^ | 5.00·10^-9^ | 1.30·10^-8^ | 3.00·10^-9^ |
| 7 | 1.61·10^-8^ | 8.85·10^-9^ | 2.80·10^-8^ | 4.00·10^-9^ | 5.80·10^-8^ | 1.00·10^-8^ |
| 8 | 7.12·10^-8^ | 2.80·10^-9^ | 9.50·10^-8^ | 4.00·10^-9^ | 1.90·10^-7^ | 1.00·10^-8^ |
| 9 | 3.03·10^-7^ | 1.28·10^-8^ | 3.20·10^-7^ | 3.00·10^-8^ | 5.90·10^-7^ | 5.00·10^-8^ |
| 10 | 6.70·10^-7^ | 8.97·10^-10^ | 9.20·10^-7^ | 4.00·10^-8^ | 9.30·10^-7^ | 3.00·10^-8^ |
| 11 | 1.15·10^-6^ | 5.34·10^-8^ | 1.13·10^-6^ | 3.00·10^-8^ | 1.15·10^-6^ | 4.00·10^-8^ |
| 12 | 1.16·10^-6^ | 1.76·10^-8^ | 1.14·10^-6^ | 3.00·10^-8^ | 1.16·10^-6^ | 4.00·10^-8^ |
